# Pro‐inflammatory cytokines as emerging molecular determinants in cardiolaminopathies

**DOI:** 10.1111/jcmm.16975

**Published:** 2021-11-12

**Authors:** Andrea Gerbino, Cinzia Forleo, Serena Milano, Francesca Piccapane, Giuseppe Procino, Martino Pepe, Mara Piccolo, Piero Guida, Nicoletta Resta, Stefano Favale, Maria Svelto, Monica Carmosino

**Affiliations:** ^1^ Department of Biosciences, Biotechnologies and Biopharmaceutics University of Bari Bari Italy; ^2^ Department of Emergency and Organ Transplantation Cardiology Unit University of Bari Aldo Moro Bari Italy; ^3^ Regional General Hospital “F. Miulli” Acquaviva delle Fonti Italy; ^4^ Division of Medical Genetics Department of Biomedical Sciences and Human Oncology University of Bari Aldo Moro Bari Italy; ^5^ Department of Sciences University of Basilicata Potenza Italy

**Keywords:** cardiolaminopathies, cytokines, inflammation

## Abstract

Mutations in Lamin A/C gene (*lmna*) cause a wide spectrum of cardiolaminopathies strictly associated with significant deterioration of the electrical and contractile function of the heart. Despite the continuous flow of biomedical evidence, linking cardiac inflammation to heart remodelling in patients harbouring *lmna* mutations is puzzling. Therefore, we profiled 30 serum cytokines/chemokines in patients belonging to four different families carrying pathogenic *lmna* mutations segregating with cardiac phenotypes at different stages of severity (*n* = 19) and in healthy subjects (*n* = 11). Regardless *lmna* mutation subtype, high levels of circulating granulocyte colony‐stimulating factor (G‐CSF) and interleukin 6 (IL‐6) were found in all affected patients’ sera. In addition, elevated levels of Interleukins (IL) IL‐1Ra, IL‐1β IL‐4, IL‐5 and IL‐8 and the granulocyte‐macrophage colony‐stimulating factor (GM‐CSF) were measured in a large subset of patients associated with more aggressive clinical manifestations. Finally, the expression of the pro‐inflammatory 70 kDa heat shock protein (Hsp70) was significantly increased in serum exosomes of patients harbouring the *lmna* mutation associated with the more severe phenotype. Overall, the identification of patient subsets with overactive or dysregulated myocardial inflammatory responses could represent an innovative diagnostic, prognostic and therapeutic tool against Lamin A/C cardiomyopathies.

## 1 INTRODUCTION

Mutations in *lmna* gene encoding intermediate filament proteins of the inner nuclear membrane Lamin A/C (LMNA) cause tissue‐specific systemic diseases collectively known as laminopathies. Cardiac laminopathies encompass a wide spectrum of clinical entities with high penetrance and (usually) adult onset. Although dilated cardiomyopathy with conduction defects (DCM‐CD) is the most prevalent phenotype, different mutational sites might correlate with different clinical manifestations spanning from conduction disorders, frequent atrial fibrillation and life‐threatening ventricular arrhythmias, with normal or altered ventricular systolic function.^1^, ^2^ This surprising large number of cardiac phenotypes reflects an even larger number of specific mutations (about 500) identified in the *lmna* gene and collected in the UMD‐*lmna* mutations database.

The molecular mechanisms underlying the origin and development of such a wide spectrum of cardiac pathologies are still incompletely defined. Despite under the spotlight of many research groups for quite a long time, a complete understanding of the cellular functions mediated by LMNA is missing. Thus, mutations in the *lmna* gene can either affect a specific function of LMNA or broadly impact the whole repertoire of molecular mechanisms controlled by these intermediate filaments such as mechanotransduction, gene regulation and signal transduction.^3^, ^4^ Yet, we still do not know whether these different pathogenic mechanisms represent different pieces of the same puzzle or each one of them can be correlated to a specific clinical entity.

For instance, alterations in several cellular pathways, including WNT/β‐catenin, mitogen‐activated protein kinases (MAPKs) and protein kinase B (AKT)/mammalian target of rapamycin (mTOR) signalling, have been identified in LMNA^H222P/H222P^ mice, a mouse model of the human LMNA‐associated DCM‐CD.^5^, ^6^ Interestingly, *lmna* mutations may also impinge the machinery involved in Ca^2+^ handling into the ER^7^, ^8^, ^9^ and the connexin 43 (CX43) expression/activity at the plasma membrane in cardiomyopathies.^10^, ^11^ More recently, Salvarini et al. demonstrated that the epigenetic inhibition of the sodium voltage‐gated channel alpha subunit 5 (SCN5A) in cardiomyocytes differentiated from IPS derived from patients with K219T‐LMNA pathogenic variant^12^ can account for the conduction defects reported in the clinical history of these arrhythmogenic patients.

Despite the molecular mechanism involved in LMNA cardiomyopathies, the deterioration in electrical and contractile function correlates with exacerbated cardiomyocytes damage or death, which, in turn, may trigger myocardial inflammation, further aggravating the progression of the cardiomyopathy. Myocardial fibrosis is also thought to be responsible for the development of both electrical instability and mechanical impairment in cardiac laminopathies^13^ typically developing within the interventricular septum, near the region of the conduction system thus accounting for the conduction disease.^14^ It is known that the mammalian heart contains a population of resident macrophages that proliferate following myocardial damage such as in myocardial infarction, which in turn recruit other monocytes to the heart, contributing to myocardial interstitial fibrosis and adverse cardiac remodelling.^15^ Moreover, it also well established that pro‐inflammatory cytokines are produced in typical inflammatory cardiomyopathies as consequence of pathogen infection and a wide variety of toxic substances, drugs and systemic immune‐mediated diseases. These cardiomyopathies evolve also in DCM and heart failure.^16^

However, in inherited cardiomyopathies, cardiac inflammation and its correlation to contractile dysfunction and cardiac remodelling have not been fully investigated. Therefore, in this study, we aim at investigating the specific profile of 30 cytokines and chemokines in the serum of four different groups of patients harbouring different pathogenic mutations in *lmna* gene and their family members not carrying the mutation. The clinical phenotype associated with the *lmna* mutations under investigation was mainly characterized by left ventricular dilation, left ventricular systolic dysfunction and conduction defects despite at different range of severity, even amongst members of the same family. The profile of inflammatory cytokines measured in our experiments did not show any specificity for the location of each mutation analysed. However, the severity of the clinical manifestations associated with each patient correlates with the degree of inflammation in terms of numbers of pro‐inflammatory cytokines upregulated. Since no specific pharmacological treatment is available and the ICD implantation is currently the only effective clinical approach for patients affected by cardiac laminopathies, the identification of patient subsets affected by LMNA cardiomyopathies with overactive or dysregulated myocardial inflammatory responses could be crucial for clarifying the pathogenesis of these cardiomyopathies and for evaluating immediate successful therapeutic approaches.

## 2 METHODS

### 2.1 Clinical and instrumental analysis

The patients who were referred to the Cardiomyopathy Unit, Cardiology Unit, Department of Emergency and Organ Transplantation, University of Bari Aldo Moro, Bari (Italy), between January 2020 and August 2020, and who fulfilled the inclusion and exclusion criteria, were involved in the study. A total of 30 Italian patients (19 patients with *lmna* mutation e and 11 *lmna* mutation‐negative family members) were enrolled in the study and subjected to blood sampling for serum cytokine assay. To avoid variations in serum levels of cytokines, no subject had exercised physical activity prior to blood sampling and did not have any ongoing infections or immunodeficiency conditions at the time of enrolment. For further details on inclusion/exclusion criteria, see Appendix S1. All recruited subjects provided their written informed consent to participate in this study. The project conformed to the principles of the Declaration of Helsinki (World Medical Association) and was approved by the Ethics Committee of the University Hospital Consortium, Policlinico of Bari, Italy.

### 2.2 Cytokine/chemokine assay

Plasma samples from the patients included in the study were prepared by centrifugation at 500 *g* for 12 min and stored at −80°C. Bio‐Plex Pro Human Cytokine 27‐plex Assay (#M500KCAF0Y; Bio‐Rad Laboratories) and Bio‐Plex Pro Transforming Growth Factor‐β (TGF‐β) 3‐plex Assay (#171W4001 M; Bio‐Rad Laboratories) were used by following the manufacturer's instructions. Each sample was analysed in triplicate in BioPlexMagpix Multiplex Reader (Bio‐Rad Laboratories), and the data automatically analysed using Bio‐Plex Manager 6.0 software (Bio‐Rad Laboratories). For the list of cytokines analysed and for detailed procedure, see Appendix S1.

### 2.3 Serum exosome preparation and analysis

Serum exosomes were isolated from 250 μl of each patients’ serum with the exosome isolation kit EXOQ50A‐1 (System Biosciences) according to manufacturer's instructions. Serum exosomes were analysed by Western blotting for the expression of Hsp70 and exosome markers. For details on serum exosomes preparation and analysis, see Appendix S1.

### 2.4 Cell culture and western blotting

HEK293 cells were transiently transfected with the previously described^17^ vectors for the expression of either LMNA WT or LMNA‐p. Leu140_Ala146dup variant, using Lipofectamine® 2000. After 72 h, transfection cells were lysed in RIPA buffer and lysate were analysed by Western blotting for the expression of Hsp70. For details, see Appendix S1.

### 2.5 Statistical analysis

Continuous variables are expressed as mean values ± standard deviation and compared between groups by using Student's *t* test (equal or unequal variance as appropriate). Categorical variables are expressed as absolute frequency or percentage. Associations were tested with Fisher's exact test. Analyses were performed using STATA software version 14 (Stata). Student's *t* test for unpaired data was used to analyse differences in cytokines levels between each patient group and controls. Receiver operating characteristic (ROC) curves were used to estimate the diagnostic potential of the quantified individual cytokines to discriminate between groups. GraphPad Prism software (version 8) was used for statistical and graphical arts. In all cases, significance was considered at *p* < 0.05.

## 3 RESULTS

### 3.1 Molecular analysis of the patients’ population

The *lmna* mutations included in this study were identified in members of Italian families with cardiac phenotypes screened in our Clinical Unit dedicated to cardiomyopathies.

The *lmna* mutations are as follows:

1. The heterozygous variant c.418_438dup consists of a duplication of 21 nucleotides (CTGCTGAACTCCAAGGAGGCC) in the exon 2 of the *lmna* gene, located in the coil 1B of the central α‐helical rod domain of the LMNA protein. The resulting LMNA variant is predicted to result in the duplication of seven amino acids (LLNSKEA) in LMNA protein, from Leucine at position 140 to Alanine at position 146, without a frame shift in the open reading frame. This LMNA variant hereinafter referred to as p.Leu140_Ala146dup has been previously identified and characterized *in vitro* by our group^17^ and classified as pathogenic.
2. The heterozygous variant c.329delG consists of a G deletion at position 329 in the exon 2 of the *lmna* gene. This deletion causes a shift in the reading frame starting at Arginine 110, changing it to a Leucine and creating a premature stop codon at position 7 of the new reading frame. This LMNA variant, denoted as p.Arg110Leufs*7, is present in the ClinVar data base and ranked as likely pathogenic.
3. The heterozygous variant c.949G>A consists of a G to A substitution at position 949 in the exon 6 of the *lmna* gene leading to glutamate to lysine exchange at the position 317 in the coil2 domain. This variant (p.Glu317Lys) is ranked as pathogenic and has been already described in patients with atrioventricular block (AVB) and DCM.^18^, ^19^
4. The heterozygous variant c.569G>A, located in coding exon 3 of the *lmna* gene, results from a G to A substitution at nucleotide position 569. The arginine at codon 190 is replaced by glutamine, an amino acid with highly similar properties. This variant is reported as p.Arg190Gln and ranked as pathogenic. This alteration has been already reported in individuals with DCM and reported as LMNA R190Q variant.^20^, ^21^

### 3.2 Clinical data

Table 1 summarizes the most relevant clinical manifestations of all the subjects evaluated in this study. Carriers of pathogenic LMNA variants belonging to four different families (patients) were compared with family members not carrying the mutation (controls). Clinical data, electrical and mechanical abnormalities documented by ECG recordings and cardiac imaging manifested during their clinical history were reported.

**TABLE 1:** Clinical characteristics of study subjects according to *lmna* mutation carrier status

	*lmna* mutation‐positive subjects according to different genotypes (*n* = 19)				All *lmna* mutation‐positive subjects (*n* = 19)	*lmna* mutation‐negative subjects (*n* = 11)	*p* value
	p. Leu140_Ala146dup (*n* = 6)	p. Arg110Leufs*7 (*n* = 4)	p. Glu317Lys (*n* = 6)	p. Arg190Gln (*n* = 3)	Patients (*n* = 19)	Controls (*n* = 11)	
Age at time of blood collection (years)	46 ± 10	42 ± 13	54 ± 6	35 ± 16	46 ± 12	41 ± 18	0.399
Gender (F/M)	4/2	3/1	2/4	0/3	9/10	7/4	0.466
Cardiac phenotype	DCM, left ventricular systolic dysfunction, AF, AVB, PVCs, NSVT, SVT	Left ventricular systolic dysfunction, AF, AVB, PVCs, NSVT	DCM, left ventricular systolic dysfunction, AF, AVB, PVCs, NSVT, SVT	DCM, Left ventricle systolic dysfunction, AF		Healthy	
Clinical myopathy	0	0	0	3	3	0	
CPK levels (U/L)	163 ± 53	112 ± 68	59 ± 31	1274 ± 1255	339 ± 655	NA	
SBP (mm Hg)	104 ± 13	106 ± 11	126 ± 20	153 ± 24	119 ± 24	118 ± 9	0.822
DBP (mm Hg)	65 ± 5	66 ± 8	83 ± 9	90 ± 13	75 ± 13	71 ± 7	0.320
BMI (Kg/m^2^)	24.9 ± 3.3	27.1 ± 6.9	23.7 ± 3.4	27.5 ± 4.3	25.4 ± 4.3	24.3 ± 2.3	0.427
Heart rate (bpm)	60 ± 7	61 ± 11	62 ± 6	57 ± 2	60 ±7	73 ± 13	**0.010**
PR interval (ms)	250 ± 62	210 ± 68	277 ± 178	167± 23	237 ± 111	147± 29	**0.003**
QRS duration (ms)	93 ± 8	89 ± 10	97 ± 10	107 ± 12	96 ±11	87 ± 10	**0.026**
QTc interval (ms)	417 ± 24	398 ± 17	421 ± 22	410 ± 47	413 ± 26	415 ± 16	0.816
AF, *n* (%)	5 (83%)	2 (50%)	3 (50%)	1 (33%)	11 (58%)	0 (0%)	**0.002**
AV block, *n* (%)	5 (83%)	2 (50%)	3 (50%)	0 (0%)	10 (53%)	0 (0%)	**0.004**
PVCs >500/24h, *n* (%)	6 (100%)	3 (75%)	2 (33.3%)	0 (0%)	11 (58%)	0 (0%)	**0.002**
PVCs >1000/24h, *n* (%)	6 (100%)	3 (75%)	1 (16.6%)	0 (0%)	10 (53%)	0 (0%)	**0.004**
NSVT, *n* (%)	6 (100%)	2 (50%)	3 (50%)	0 (0%)	11 (58%)	0 (0%)	**0.002**
SVT/VF, *n* (%)	3 (50%)	0 (0%)	2 (33%)	0 (0%)	5 (26%)	0 (0%)	0.129
Indexed LVEDV (ml/m^2^)	74 ± 16	66 ± 12	90 ± 7	86 ± 26	78 ± 17		
LVEDD (mm)	52 ± 6	48± 4	53 ±9	53 ± 5	52 ± 6	44 ± 3	**<0.001**
LVEF (%)	45 ± 13	53 ± 6	41 ± 15	56 ± 5	47 ± 12	59 ± 3	**0.001**
CMRI, *n* (%)	5 (83%)	2 (50%)	3 (50%)	2 (66%)	12 (63%)	/	
LGE on CMRI, *n* (%)	4 (80%)	0 (0%)	1 (33%)	0 (0%)	5 (42%)	/	
PM/ICD implantation, *n* (%)	5 (83%)	2 (50%)	2 (33%)	1 (33%)	9 (47%)	/	
Heart transplant, *n* (%)	1 (17%)	0 (0%)	0 (0%)	0 (0%)	1 (5%)	/	

Note

Mean ± Standard Deviation.

Abbreviations: AF, atrial fibrillation; AVB, atrioventricular block; BMI, body mass index; CMRI, cardiac magnetic resonance imaging; DBP, diastolic blood pressure; DCM, dilated cardiomyopathy; ICD, implantable cardioverter‐defibrillator; LGE, late gadolinium enhancement; LVEDD, left ventricular end‐diastolic diameter; LVEDV, left ventricular end‐diastolic volume; LVEF, left ventricular ejection fraction; NSVT, non‐sustained ventricular tachycardia; PM, pacemaker; PVCs, premature ventricular complexes; SBP, systolic blood pressure; SVT, sustained ventricular tachycardia; VF, ventricular fibrillation.

*p*‐values ≤ 0.05 were considered statistically significant and reported in bold.

#### 3.2.1 Family 1

**FIGURE 1:**
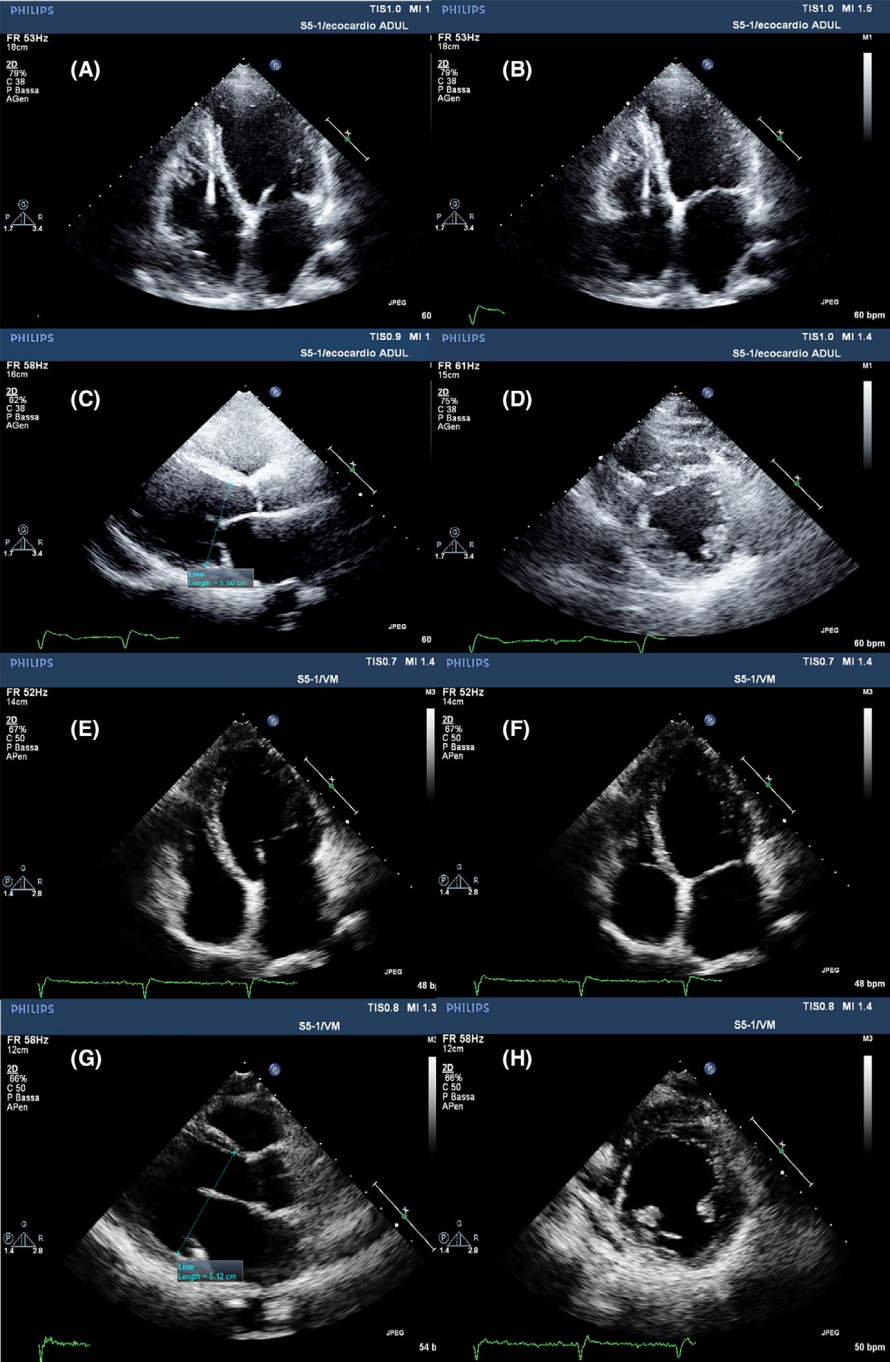
Cardiac phenotype evaluated by Echocardiographic imaging in LMNA mutants’ carriers. The upper half of the image shows LMNA DCM phenotype: (A) and (B) panels show apical four chamber (4C) views with chamber dilatation (LVEDVi 82 ml/m^2^ and LVESVi 52 ml/m^2^) and reduced left ventricular systolic function (LVEF 35%) due to global hypocontractility. (C) PLAX view: LVEDD (55 mm). (D) PSAX view: mid‐LV at the level of papillary muscles. The lower part (panels E–H) of the picture shows a hypokinetic non‐dilated LMNA cardiomyopathy characterized by normal ventricular volumes both in end‐diastole (E) (LVEDVi 64 ml/m^2^) and end‐systole (F) (LVESVi 38.8 ml/m^2^) with moderate systolic left ventricular dysfunction (LVEF 36%). (G) In PLAX view: LVEDD (51 mm). (H) PSAX view: mid‐LV at the level of papillary muscles. DCM, dilated cardiomyopathy; LVEDD, left ventricular end‐diastolic diameter; LVEDV, left ventricular end‐diastolic volume index; LVEF, left ventricular ejection fraction; LVESVi, left ventricular end‐systolic volume index; PLAX view, parasternal long‐axis view; PSAX, parasternal short‐axis view

**FIGURE 2:**
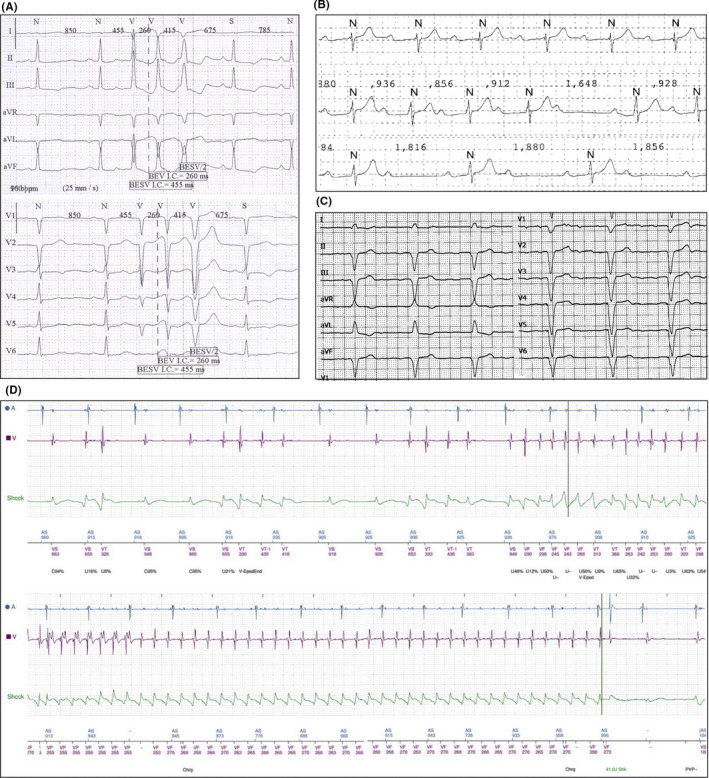
Abnormal ECG findings in LMNA mutants’ carriers. (A) Episode of non‐sustained VT with LBBB morphology and inferior axis on 12‐lead Holter monitoring. (B) ECG showing sinus rhythm, first‐degree atrioventricular block (upper trace), type 1 s‐degree atrioventricular block (middle trace) and 2:1 atrioventricular block (lower trace) recorded in the same patient during 24‐h ECG recording. (C) 12‐lead ECG displaying atrial fibrillation and ventricular rhythm induced by pacemaker with VVI pacing mode. (D) ICD remote monitoring report showing PVCs which trigger an episode of sustained TV, terminated, after ineffective ATP, by internal ICD shock. ATP, antitachycardia pacing; ICD, implantable cardioverter‐defibrillator; LBBB, left bundle branch block; PVCs, premature ventricular complexes; VT, ventricular tachycardia

Patients harbouring p. Leu140_Ala146dup LMNA variant mostly exhibited (4 out of 6) a phenotype characterized by left ventricular dilation (LVEDD 52 ± 6 mm; LVEDVi 74 ± 16 ml/m^2^), left ventricular systolic dysfunction (LVEF 45 ± 13%) (Figure 1A–D) and numerous arrhythmic disorders. Indeed, frequent (up to 1000 per day) polymorphic premature ventricular complexes (PVCs), and non‐sustained ventricular tachycardia (NSVT) episodes were recorded on ECG Holter monitoring in all patients of this group (Figure 2A); three patients also showed sustained ventricular tachycardia (SVT) or ventricular fibrillation (VF) events, detected on telemetry or implantable cardioverter‐defibrillator (ICD) arrhythmia registry. Conduction disturbances (first‐, second‐ and third‐degree atrioventricular blocks, Figure 2B) and atrial fibrillation (Figure 2C) were observed in about 80% of the patients. Five patients received an ICD in primary prevention, and several device appropriate interventions by antitachycardia pacing (ATP) or DC shock on SVT or fast SVT recognized in VF zone, respectively, were detected in three of them (Figure 2D). Finally, a patient of this group underwent heart transplant (after unsuccessful VT trans‐catheter ablation) at the age of 45 due to sustained VT recurrences in storm and subsequent LV function deterioration (LEVF 30%).^17^

#### 3.2.2 Family 2

The patients of this family, with the p. Arg110Leufs*7 LMNA pathogenic variant, showed a less aggressive clinical phenotype in comparison with members of the previously described family. In these subjects, ventricular dimensions were in the normal range (LVEDD 48 ± 4 mm; LVEDVi 66 ± 12 ml/m^2^) and only mild left ventricular systolic dysfunction was observed (LVEF 53 ± 6%). Half of the carriers developed conduction disturbances (first‐degree AV block), atrial fibrillation and ventricular arrhythmias as NSVT episodes, and many PVCs were recorded at the 24‐hour Holter monitoring. Two subjects underwent ICD implantation in primary prevention, and one of them, with moderate left ventricular systolic dysfunction (LVEF 45%), underwent up‐grading of dual‐chamber pacemaker to biventricular ICD with subsequent recovery of the left ventricular systolic function. To date, no members of this family presented life‐threatening ventricular tachyarrhythmias. Furthermore, two patients carrying the above variant showed neuromuscular involvement (radiculopathy associated with motor block, waddling gait and girdle hypertrophy) still undergoing diagnostic definition.

#### 3.2.3 Family 3

Cardiac phenotype of p. Glu317Lys LMNA variant carriers was characterized by left ventricular dilatation (LVEDD 53 ± 9 mm; LVEDVi 90 ± 7 ml/m^2^) and systolic dysfunction (LVEF 41 ± 15%). Three patients also displayed conduction disorders: 2 of them presented first‐degree AV‐blocks with very long PR intervals (the longest reported was 520 ms), while the third patient developed high‐degree AV block and received a dual‐chamber pacemaker subsequently upgraded to a biventricular defibrillator. Atrial fibrillation and NSVT were found in 50% of these patients, while 2 patients also presented with SVT (one of them occurring during exercise testing, and the other was detected on defibrillator arrhythmia registry). One patient received an ICD in primary prevention due to severe left ventricular dysfunction (LVEF 20%), which subsequently progressively improved (last reported LVEF was 47%) after optimized drug treatment for heart failure, and the occurrence of several appropriate defibrillator interventions by ATP on SVT was also reported.

#### 3.2.4 Family 4

The p. Arg190Gln LMNA variant was related to neuromuscular involvement consisting of muscle cramps, reduced strength to stress and inappropriate muscular hypertrophy, associated with significantly increased CPK values (1274 ± 1255 U/L), in addition to cardiac abnormalities. One of the variant carriers had left ventricular dilatation with normal ventricular function, while his brother showed a hypokinetic non‐dilated cardiomyopathy phenotype with mildly impaired left ventricular systolic function (LVEF 50%) and normal ventricular volumes (Figure 1E–H); thus far, none of these two patients has developed ventricular and supraventricular arrhythmias or conduction disturbances. The third member of this family, the father, having normal left ventricular size and systolic function, showed paroxysmal atrial fibrillation with low ventricular rate and underwent ICD implantation in primary prevention due to sinus node disease and a wide QRS tachycardia episode occurrence during exercise testing.

All *lmna* mutation‐negative family members were clinically asymptomatic, and none of them showed LMNA‐linked cardiac phenotypes either in terms of electrical disorders or mechanical abnormalities (Table 1).

### 3.3 Cytokine and chemokine levels in the sera of the LMNA patients and comparison with those of healthy controls

Sera from the patients and controls described in the Table 2 were screened for the circulating levels of 30 cytokines/chemokines (Table 2). The levels of IL‐15 are under the lower limit of the assay sensitivity, thus resulted undetectable in our cohort of patients. This is in line with the fact that it has been reported that in humans, the levels of circulating IL‐15 under normal conditions are low or undetectable (~1 pg/ml).^22^

**TABLE 2:** Serum levels of chemokines/cytokines expressed in pg/ml. Only the *p* values of significative differences compared with controls were indicated (in bold). OOR< indicates that cytokine levels are under the lower limit of the assay sensitivity

	p. Leu140_Ala146dup (*n* = 6)	p. Arg110Leufs*7 (*n* = 4)	p. Glu317Lys (*n* = 6)	p. Arg190Gln (*n* = 3)	Controls (*n* = 11)
IL‐1β	6.530 ± 1.522 ***p* = 0.0011**	3.715 ± 1.937 *p* = 0.56	4.918 ± 1.361 ***p* = 0.01**	2.643 ± 0.6233 *p* = 0.5	1.849 ± 0.2817
IL‐1ra	398.4 ± 30.03 ***p* = 0.001**	279.5 ± 14.29 ***p* = 0.0016**	396.0 ± 39.83 ***p* = 0.0002**	213.9 ± 82.23 *p* = 0.22	132.4 ± 23.15
IL‐2	9.259 ± 2.244 *p* = 0.5608	8.193 ± 2.069 *p* = 0.7	12.95 ± 4.312 *p* = 0.19	8.237 ± 1.411 *p* = 0.74	7.698 ± 0.8279
IL‐4	9.680 ± 1.680 ***p* = 0.0005**	4.578 ± 1.091 *p* = 0.52	7.948 ± 3.129 *p* = 0.16	4.523 ± 0.8520 *p* = 0.53	3.343 ± 0.4856
IL‐5	20.99 ± 4.100 ***p* = 0.0004**	9.923 ± 1.691 *p* = 0.07	31.84 ± 11.32 ***p* = 0.0064**	18.76 ± 10.69 ***p* = 0.05**	5.679 ± 1.150
IL‐6	5.82 ± 1.0 ***p* = 0.004**	7.195 ± 1.805 ***p* = 0.003**	11.79 ± 3.19 ***p* = 0.001**	6.657 ± 1.764 ***p* = 0.004**	2.743 ± 0.3996
IL‐7	39.71 ± 5.560 *p* = 0.1554	29.17 ± 4.482 *p* = 0.78	35.56 ± 3.523 *p* = 0.31	30.71 ± 8.330 *p* = 0.99	30.68 ± 2.903
IL‐8	75.39 ± 18.43 *p* = 0.7383	198.8 ± 35.50 ***p* = 0.005**	256.5 ± 41.46 ***p* = 0.0003**	216.5 ± 36.85 ***p* = 0.003**	83.73 ± 16.44
IL‐9	140.8 ± 6.288 *p* = 0.4683	139.1 ± 6.032 *p* = 0.58	169.2 ± 14.11 *p* = 0.44	141.0 ± 11.05 *p* = 0.68	152.4 ± 13.69
IL‐10	2.684 ± 0.6863 *p* = 0.9671	1.677 ± 0.7888 *p* = 0.49	2.564 ± 1.309 *p* = 0.97	2.523 ± 0.6584 *p* = 0.93	2.633 ± 1.020
IL‐12(p70)	6.952 ± 0.9793 *p* = 0.5925	6.720 ± 1.217 *p* = 0.7	9.263 ± 1.709 *p* = 0.07	11.06 ± 4.952 *p* = 0.10	6.298 ± 0.7212
IL‐13	4.295 ± 0.8268 *p* = 0.6949	3.093 ± 0.3060 *p* = 0.67	5.575 ± 0.9616 *p* = 0.24	3.083 ± 0.8539 *p* = 0.72	3.785 ± 0.9604
IL‐15	OOR<	OOR<	OOR<	OOR<	OOR<
IL‐17A	27.16 ± 5.176 *p* = 0.1860	24.26 ± 6.430 *p* = 0.34	39.71 ± 14.88 *p* = 0.09	24.65 ± 3.600 *p* = 0.21	20.02 ± 1.597
Eotaxin	177.8 ± 29.02 *p* = 0.3068	138.6 ± 21.04 *p* = 0.94	178.0 ± 22.04 *p* = 0.26	130.9 ± 38.26 *p* = 0.81	141.2 ± 20.28
bFGF	54.50 ± 6.378 *p* = 0.3689	52.57 ± 7.433 *p* = 0.49	72.84 ± 16.73 *p* = 0.08	56.65 ± 6.106 *p* = 0.19	48.02 ± 2.953
G‐CSF	2980 ± 523.8 ***p* = 0.0001**	1486 ± 442.6 ***p* = 0.0035**	1140 ± 220.4 ***p* = 0.002**	1398 ± 188.5 ***p* = 0.0001**	340.6 ± 57.89
GM‐CSF	6.250 ± 1.060 ***p* = 0.0005**	3.067 ± 1.011 *p* = 0.19	5.355 ± 0.9873 *p* = 0.05	5.473 ± 2.787 *p* = 0.06	1.978 ± 0.3146
IFN‐γ	14.97± 5.036	10.33±0.8819	11.35± 1.888	12.45± 5.019	11.47± 4.499
IP‐10	748.8 ± 52.22 *p* = 0.0650	610.3 ± 105.9 *p* = 0.84	1030 ± 285.7 *p* = 0.08	402.4 ± 53.65 *p* = 0.13	587.4 ± 63.58
MCP‐1 (MCAF)	37.97 ± 4.896 *p* = 0.1871	31.78 ± 3.089 *p* = 0.10	73.28 ± 15.05 *p* = 0.11	37.91 ± 5.729 *p* = 0.35	48.88 ± 6.413
MIP‐1α	21.52 ± 8.443 *p* = 0.4025	33.59 ± 14.43 *p* = 0.08	29.08 ± 6.912 *p* = 0.06	40.18 ± 1.161 ***p* = 0.005**	14.02 ± 3.919
PDGF‐bb	3233 ± 360.7 *p* = 0.2717	3388 ± 177.8 *p* = 0.52	3851 ± 394.6 *p* = 0.96	4797 ± 332.4 *p* = 0.31	3883 ± 437.6
MIP‐1b	148.5 ± 35.34 *p* = 0.3511	142.2 ± 23.39 *p* = 0.28	155.5 ± 22.78 *p* = 0.10	108.4 ± 10.58 *p* = 0.82	114.0 ± 12.52
RANTES	11929 ± 556.9 *p* = 0.1784	12446 ± 421.6 *p* = 0.51	14188 ± 518.9 *p* = 0.711	11606 ± 1082 *p* = 0.35	13630 ± 1043
TNF‐γ	99.09 ± 29.64 *p* = 0.2234	80.49 ± 21.18 *p* = 0.23	95.85 ± 20.49 *p* = 0.05	64.04 ± 7.893 *p* = 0.92	63.16 ± 4.318
VEGF	108.2 ± 47.78 *p* = 0.5554	209.5 ± 86.52 *p* = 0.15	133.4 ± 47.72 *p* = 0.34	161.0 ± 43.49 *p* = 0.14	72.98 ± 29.99
TGF‐β1	49020 ± 2220 *p* = 0.6832	54484 ± 476.7 *p* = 0.9888	51638 ± 4105 *p* = 0.9959	63726 ± 3358 *p* = 0.1202	52788 ± 2577
TGF‐β2	3169 ± 75.88 *p* = 0.9999	3282 ± 65.51 *p* = 0.8934	3421 ± 172.1 *p* = 0.1968	3244 ± 186.3 *p* = 0.9827	3173 ± 35.17
TGF‐β3	1627 ± 23.58 *p* = 0.6128	1666 ± 57.12 *p* = 0.9790	1548 ± 105,3 *p* = 0.1534	1770 ± 83.44 *p* = 0.9204	1705 ± 38.60

Abbreviations: IL, interleukin; bFGF, basic fibroblast growth factor; G‐CSF, granulocyte colony‐stimulating factor; GM‐CSF, granulocyte‐macrophage colony‐stimulating factor; IFN‐γ, γ‐interferon; IP‐10, interferon gamma‐induced protein‐10; MCP‐1, monocyte chemoattractant protein‐1; MIP‐1α, MIP‐1β, macrophage inflammatory protein‐1α and 1β; PDGF‐BB, platelet‐derived growth factor‐BB; TNF‐ α, tumour necrosis factor‐α; VEGF, vascular endothelial growth factor; TGF‐β, transforming growth factor‐β.

*p*‐values ≤ 0.05 were considered statistically significant and reported in bold.

The levels of several pro‐inflammatory cytokines resulted upregulated in the sera of patients compared with controls. A more detailed analysis of these cytokines was performed.

**FIGURE 3:**
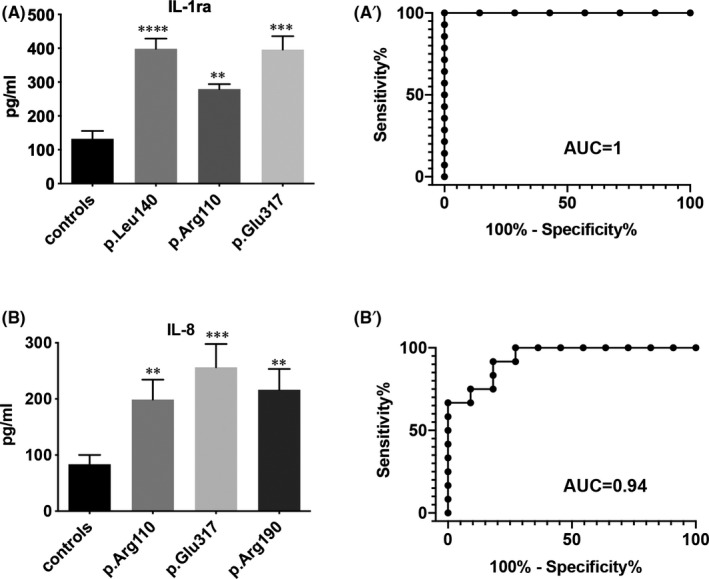
Serum levels of IL1‐ra (A) and IL‐8 (B) in the family enrolled in the study and in healthy controls. **p < .01; ***p < .001. Areas under the curves (AUC) obtained with the sensitivity and specificity for the serum levels of IL1‐ra (A’) and IL‐8 (B’) in patients with LMNA mutant shown in A and in B respectively

As shown in Figure 3A and Table 2, IL‐1ra resulted significantly upregulated in the serum of both p. Leu140_Ala146dup, p. Arg110Leufs*7 and p. Glu317Lys carriers compared with controls (*p* = 0.0001, *p* = 0.0016 and *p* = 0.0002 respectively). The area under the ROC curve (AUC) for IL‐1ra was 1 (Figure 3A’, *p* = 0.0003), suggesting that the measurement of the levels of this cytokine might allow us to discriminate between patients carrying either p. Leu140_Ala146dup, p. Arg110Leufs*7 or p. Glu317Lys LMNA variants and controls enrolled in our study with high degree of accuracy. Moreover, levels of IL‐8 resulted significantly increased in the serum of p. Arg110Leufs*7, p. Glu317Lys and p. Arg190Gln LMNA carriers compared with controls (Figure 3B, Table 2). The AUC values are 0.93 ± 0.04 (Figure 3B’, *p* = 0.0004), indicating that IL‐8 levels may be also considered as accurate biomarkers for the cardiomyopathy in p. Arg110Leufs*7, p. Glu317Lys and p. Arg190Gln carriers.

**FIGURE 4:**
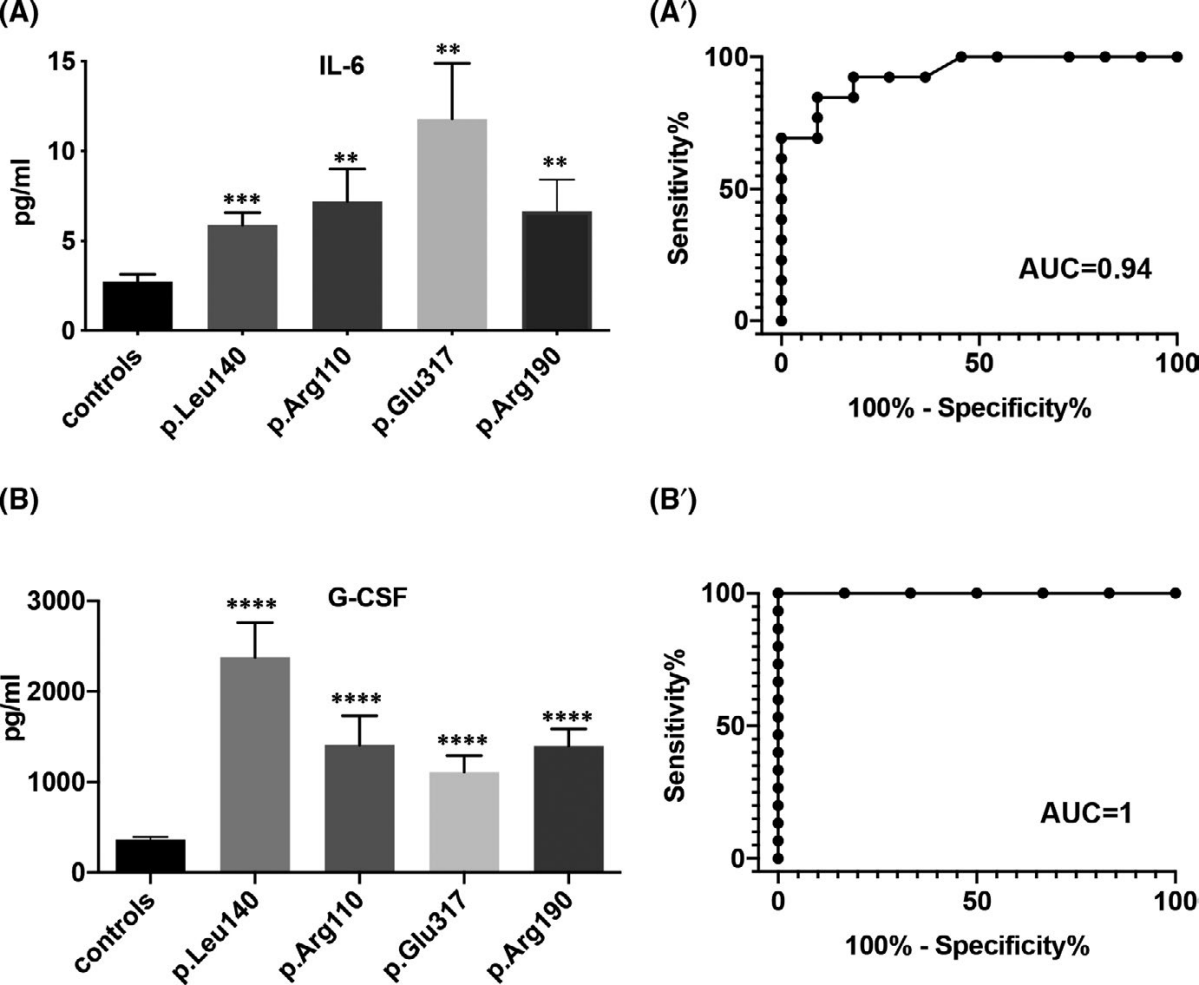
Serum levels of IL6 (A) and G‐CSF (B) in the family enrolled in the study and in healthy controls. **p < .01; ***p < .001; ****p < .0001. Areas under the curves (AUC) obtained with the sensitivity and specificity for the serum levels of IL6 (A’) and G‐CSF (B’) in patients with LMNA mutant shown in A and in B, respectively

Interestingly, the levels of both IL‐6 and G‐CSF were significantly increased in all patients compared with controls (Figure 4A, B, Table 2) with the AUC value of 0.94 ± 0.04 (Figure 4A’, *p* = 0.0003) and 1 (Figure 4B’, *p* < 0.0001), respectively, indicating the levels of these cytokines as valuable parameters to distinguish between patients affected by LMNA‐associated cardiomyopathy and controls at least in our cohort of 30 individuals analysed.

**FIGURE 5:**
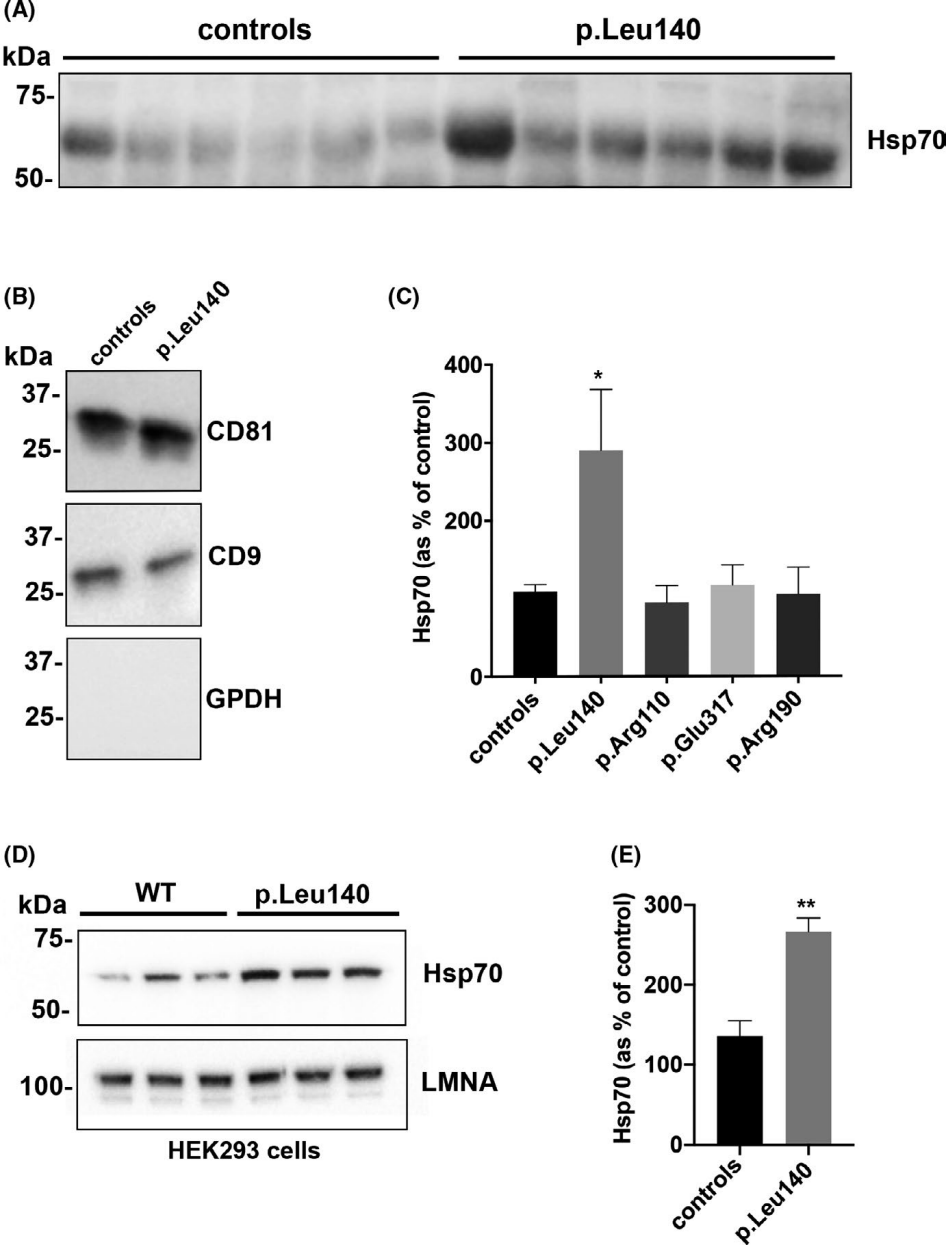
Serum levels of IL‐1β, IL‐4, IL‐5 and GM‐CSF in some of the family enrolled in the study and in healthy controls. **p < .01; ***p < .001

Serum levels of IL‐1β, IL‐4, IL‐5 and GM‐CSF were also significantly upregulated in some of the families enrolled in the study compared with controls (Figure 5). Interestingly, these cytokines were more significantly upregulated in p. Leu140_Ala146dup carriers suggesting a more complex pattern of inflammation in these patients. Interestingly, amongst the cohort of p. Leu140_Ala146dup carriers, we identified a 57‐year‐old patient who underwent heart transplantation in 2017 and asymptomatic at the time of the analysis. In this carrier, the serum levels of cytokines were comparable to that of control healthy subjects, thus suggesting a clear correlation between the clinical manifestation of the cardiomyopathy and the increased levels of pro‐inflammatory cytokines (not shown).

### 3.4 Analysis of Hsp70 in serum exosomes of LMNA mutant carriers

It has been reported that extracellular Hsp70 may act through surface receptors stimulating release of pro‐inflammatory cytokines^23^ and that elevated serum levels of Hsp70 correlate with hypertrophy and fibrosis in cardiovascular diseases.^24^

Indeed, to investigate more in deep the molecular mechanisms involved in the establishment of the inflammatory phenotype in LMNA mutant carriers, we analysed the expression of Hsp70 in serum exosomes of both patients and controls involved in the study.

**FIGURE 6:**
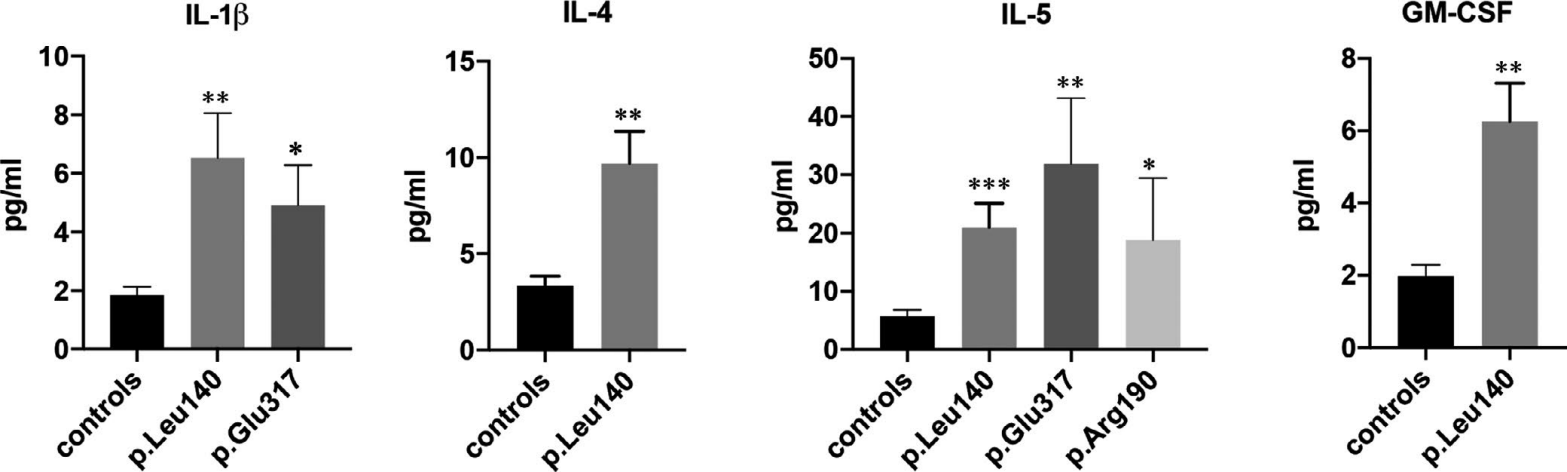
Hsp70 expression in patients’ serum exosomes and in LMNA‐p. Leu140_Ala146dup‐ expressing cells. (A) Western blotting on serum exosomes from LMNA‐p. Leu140_Ala146dup‐carrying patients (p. Leu140) and healthy controls (controls) with anti‐Hsp70 antibodies. Equal volumes of sera from each patient were loaded. (B) Western blotting on serum exosomes from LMNA‐p. Leu140_Ala146dup‐carrying patients (p. Leu140) and healthy controls (controls) with anti‐CD81, anti‐CD‐9 antibodies used as markers of exosomes purity. GAPDH absence is also index of exosome purity and was included in the panel. (C) Densitometric analysis of Hsp70‐immunoreactive bands in serum exosomes from all patients and controls included in the study. The data are means of 3 independent experiments. *p < .05. (D) Western blotting of Hsp70 and LMNA in either LMNA WT or LMNA‐p. Leu140_Ala146dup‐expressing HEK293 cells. (E) Densitometric analysis of Hsp70‐immunoreactive bands in either LMNA WT or LMNA‐p. Leu140_Ala146dup‐expressing HEK293 cells. The data are means of 3 independent experiments. **p < .01

We found an increase in the Hsp70 expression in serum exosomes from LMNA‐p. Leu140_Ala146dup carriers compared with controls (Figure 6A). The same exosomes were tested for the expression of the exosome’ markers, CD81 and CD9 (Figure 6B). Densitometric analysis of Hsp70 band in the serum of all patients and controls showed that only the circulating levels of Hsp70 were significantly upregulated in LMNA‐p. Leu140_Ala146dup carriers compared with controls (Figure 6C).

To corroborate the relationship between the elevated serum levels of Hsp70 in serum exosomes from LMNA‐p. Leu140_Ala146dup carriers and the specific LMNA mutant, we analysed the expression levels of Hsp70 in HEK293 cells after LMNA‐p. Leu140_Ala146dup expression. As shown in Figure 6D and E, the expression levels of Hsp70 increased by about twofold in LMNA‐p. Leu140_Ala146dup compared with LMNA‐WT‐expressing HEK293 cells.

## 4 DISCUSSION

In this work, we found that specific pro‐inflammatory cytokines resulted upregulated in a cohort of patients affected by cardiomyopathy due to different mutations in *lmna* gene.

Carriers expressing the pathogenic LMNA variant are characterized by bradycardia, AF, a significant increase in PR interval and QRS duration, high frequency of AV block, PVCs and NSVT occurrence, an increase in LVEDD and a decrease in LVEF compared with controls, respectively (Table 1).

The PR interval duration >200 ms and a QRS complex prolongation (Table 1) denote the presence of AV‐conduction blocks and an impaired electrical conduction within the ventricles in LMNA variant carriers. In addition, high frequency of PVCs and NSVT in LMNA mutant carriers clearly demonstrates the presence of early depolarizations originating in the ventricles of these patients due to increased ventricular automaticity. Both PVCs and NSVT are associated with an overall increased risk for clinically relevant heart failure and an increased risk for death.^25^ Moreover, LMNA mutant carries recruited in this study are characterized by mean increase in LVEDD and a decrease in LVEF compared with controls denoting a left ventricular dilation and dysfunction.

We found high levels of circulating G‐CSF and IL‐6 in all patients, and elevated levels of IL‐1ra and IL‐8 in a large subset of the patients enrolled in the study.

In particular, high levels of both G‐CSF and IL‐6 were observed in all the families we examined, independently from the mutation type and the phenotypes detected in each family. On the base of these findings, it is possible to hypothesize that high levels of both G‐CSF and IL‐6 could be associated with cardiolaminopathies. On the contrary, high values of IL‐1ra are present in three (Families 1, 2 and 3) of the investigated families whose members carrying the LMNA variant displayed a high percentage of AV‐blocks, NSVT and PVCs, which are completely absent from patients of the fourth family and from control subjects (Table 1). In addition to this, the observed PR intervals were significantly longer in patients from Families 1, 2 and 3 when compared either to patients from the fourth family or subjects not carrying LMNA variants (Table 1). Interestingly, the members of Family 4 were characterized by a different laminopathic phenotype than those belonging to the other three families, because they displayed a clear neuromuscular involvement associated with high CPK values and less severe electrical and mechanical cardiac damage. This suggests that IL‐1ra could be a biomarker associated with conduction defects and arrhythmic manifestations in LMNA patients carrying an overt cardiac phenotype.

G‐CSF is produced by bone marrow stromal cells, endothelial cells, macrophages and fibroblasts. Its production is induced by inflammatory stimuli such as pro‐inflammatory cytokines (TNF‐α, IL‐6 and IL‐1), and it may enhance the pro‐inflammatory responses by controlling neutrophil numbers and their activity during inflammation. Although we did not find any significant increase in TNF‐α in LMNA mutant carriers, we did measure a significant increase in the serum levels of IL‐1ra in p. Leu140_Ala146dup, p. Arg110Leufs*7 and p. Glu317Lys carriers and in the serum levels of IL‐6 in all LMNA mutants carriers compared with controls. IL‐1ra is a receptor antagonist of IL‐1 activity, and it is released rapidly in the circulation under the same inflammatory conditions that stimulate IL‐1α and IL‐1β. Of note, the measurement of IL‐1ra levels rather than IL‐1α or IL‐1β is a more reliable parameter of an increase in production of IL‐1 family members in inflammatory conditions since IL‐1α and IL‐1β lack the secretory peptide signal, and thus, they are not readily secreted into the systemic circulation.

IL‐1 family members can act on cardiac resident macrophages, neutrophils and parenchymal cells to trigger production of IL‐6, G‐CSF and other inflammatory mediators. It has been reported that IL‐1 is consistently upregulated in experimental models of heart failure due to a wide range of aetiologies, including myocardial infarction and diabetic cardiomyopathy.^26^ Moreover, there are experimental evidence supporting the role of IL‐1 signalling in the pathogenesis of cardiac dysfunction and adverse remodelling associated with heart failure. For instance, KO mice for IL‐1 receptors showed attenuated adverse remodelling after myocardial infarction, exhibiting suppressed inflammatory responses.^27^ IL‐1 suppresses systolic cardiomyocyte function by the disruption of calcium handling^28^ and by promulgating cardiomyocyte apoptosis.^29^ In agreement with these experimental evidence, our data support the above pathogenetic mechanisms because we found high levels of IL‐1β in Families 1 and 3. In particular, these patients displayed significantly lower LVEF values and higher prevalence of serious arrhythmic events in comparison with patients from other families (Table 1). Both left ventricular dysfunction and arrhythmias seem to be associated with IL‐1β increased levels in our patients, thus potentially confirming the role of this cytokine as a biomarker related to more severe cardiac mechanical and electrical abnormalities in LMNA patients. Interestingly, IL‐1ra and G‐CSF levels were found upregulated in patient cohort affected by striated muscle laminopathies, including LMNA cardiomyopathy,^30^ thus suggesting not only a link of these cytokines with cardiac dysfunction but also laminopathies in general.

The role of IL‐6 in the pathogenesis of cardiac disease is well established in experimental models and in humans. IL‐6 is consistently upregulated in experimental models of cardiac injury and heart failure regardless of the underlying aetiology and is expressed by cardiomyocytes, infiltrating mononuclear cells and fibroblasts.^31^, ^32^ In cardiomyocytes, IL‐6 is able to decrease intracellular Ca^2+^ transients and depress cell contraction through a nitric oxide (NO)‐cGMP‐mediated pathway,^33^, ^34^ and in fibroblasts, IL‐6 promotes proliferation and stimulates extracellular matrix synthesis.^34^ Moreover, in *in vivo* studies, infusion of IL‐6 caused hypertrophy and fibrosis, and increased myocardial stiffness in mice.^35^

Importantly, high plasma levels of IL‐6 can provide prognostic information in patients with chronic heart failure (CHF), independently of ventricular dysfunction and of aetiology, suggesting an important role for IL‐6 in the pathophysiology of HF.^36^, ^37^ Regardless of specific aetiology and organ localization, systemic inflammation, via IL‐6 elevation, rapidly induces atrial electrical remodelling and electrical instability thus increasing susceptibility to atrial fibrillation, by downregulating cardiac connexins.^38^ Emerging experimental evidence suggests that IL‐6 may play a critical role in contributing to the modulation of *ICa*, *L* and *IK* currents, and both factors are active contributors to cardiac instabilities.^39^ In accordance with these experimental findings, atrial fibrillation occurrence was documented in all evaluated LMNA families.

IL‐8, which also resulted upregulated in most of the patients involved in this study, has been shown to be induced in the failing myocardium,^40^ and it was reported to predict the development of left ventricular dysfunction and the following HF together with IL‐6.^41^

Interestingly, Family 4 with a neuromuscular phenotype shows circulating levels MIP‐1α significantly higher than those from other family patients or control subjects. The association between high expression of this biomarker and neuromuscular phenotype in LMNA variant patients is consistent with data from Cappelletti C et al. who examined the cytokine profile in patients with striated muscle laminopathies.^30^ Thus, MIP‐1α could be associated with skeletal muscle involvement in cardiac laminopathy patients.

In addition to the cytokines discussed so far, IL‐4, IL‐5 and GM‐CSF resulted also significantly increased in the serum of some patients enrolled in the study.

A recent and elegant study demonstrated the cooperative role of IL‐5 and IL‐4 in the development of inflammatory dilated cardiomyopathy (DCMi). Transgenic mice overexpressing IL‐5 developed eosinophil infiltration and a severe spontaneous cardiac enlargement. The role of IL‐5 in the pathogenesis of DCMi was to mobilize IL‐4‐producing eosinophils into the myocardium, which then in turn were responsible for the dilated cardiomyopathy.^42^ Accordingly, several studies have shown a positive correlation between systemic IL‐4 levels and cardiac fibrotic remodelling and dilation in both patients and experimental animals.^43^, ^44^, ^45^

Interestingly, p. Leu140_Ala146dup carriers with the widest spectrum of pro‐inflammatory circulating cytokines dysregulated show the most severe cardiac phenotype in our cohort of LMNA mutant carriers. Notably, IL‐4 and GM‐CSF resulted to be highly expressed only in patients belonging to the above family. Since this family displayed the most severe cardiac phenotype, both in terms of cardiac function impairment and in relationship with malignant arrhythmic events occurrence, this further supports the hypothesis that the pro‐inflammatory cytokines we found upregulated in our study, significantly contribute to pathogenesis of the LMNA cardiomyopathy at least in our cohort of LMNA mutant carriers.

To gain more insights on the molecular events involved in the inflammatory response in our patients, we paid attention to the heat shock proteins since some of them have been shown to be potent activators of the innate immune system.^46^

Of note, we found significantly elevated expression levels of Hsp70 in the serum exosomes of LMNA‐p. Leu140_Ala146dup carriers and a significant increase in Hsp70 upregulation in LMNA‐p. Leu140_Ala146dup‐expressing cells. It has been reported that Hsp70 is released from cardiomyocytes undergoing lysis, necrosis and apoptosis, as well as *via* active secretion in response to a variety of stress stimuli, including ischaemia and oxidative stress.^47^, ^48^ Accordingly, we previously reported that LMNA‐p. Leu140_Ala146dup‐expressing cardiomyocytes have decreased nuclear stability, resulting in a higher rate of apoptosis.^17^ High levels of circulating Hps70 have been detected in patients with acute myocardial infarction correlating with the extent of myocardial damage.^49^ Moreover, GM‐CSF has been reported to significantly increases the expression of Hsp70 in infarcted myocardium in mice.^50^ Indeed, the most severe cardiac phenotype and the upregulated levels of GM‐CSF found only in LMNA‐p. Leu140_Ala146dup carriers may account for the elevated levels of circulating Hsp70 in these patients.

Of note, Hsp70 and IL‐6 are potential new therapeutic targets for this subset of cardiolaminopathies.

In fact, a significant cardioprotective effect of the anti‐Hsp70 blocking antibody has been demonstrated in an HF mouse model, with resolution of myocardial inflammation, left ventricular dilation and dysfunction and a significant inhibition of cardiac fibrosis.^51^ Moreover, Hsp70 blocking improves cardiac functional recovery in mice after global ischaemia reperfusion and reduces expression of the pro‐inflammatory cytokines TNF‐α, IL‐1β and IL‐6.^52^ In the clinical practice, an anti‐IL6 receptor antibody (tocilizumab) is widely used to treat the abnormal inflammatory response that occurs in autoimmune diseases as rheumatoid arthritis and it is well tolerated by patients.^53^ In addition, its in vivo administration to the mouse model of progeria significantly ameliorates the progeroid phenotype, including cardiac histology in these mice.^54^ Of note, the anti‐IL‐6 receptor antibody (MR16‐1) prevents the development of LV remodelling after MI in mice.^55^

## 5 CONCLUSIONS

Clinical course of cardiac laminopathies is characterized by a poor prognosis and a high rate of major cardiac events. So far, therapeutic approaches are exclusively symptomatic. Improvement in therapeutic management might come from very early treatment with drugs hopefully already used in clinical practice. In this scenario, we believe that our data are of great interest on the translational point of view. The main finding of our study is that inflammatory cytokines could significantly contribute to the pathogenesis of the cardiomyopathy in *lmna* mutation carriers and correlate with the severity of the cardiac phenotype. Indeed, early identification of dysregulated pro‐inflammatory serum cytokines in LMNA‐cardiomyopathy patients could be crucial for therapeutic approaches able to counteract the progression of the disease and to finally improve the prognosis of this subset of severe cardiomyopathies.

## CONFLICT OF INTEREST

The authors have no conflict of interest to declare.

## AUTHOR CONTRIBUTIONS

**Andrea Gerbino:** Conceptualization (equal); Data curation (equal); Investigation (equal); Writing‐original draft (equal). **Cinzia Forleo:** Data curation (equal); Methodology (equal); Resources (equal); Writing‐original draft (equal). **Serena Milano:** Data curation (equal); Formal analysis (supporting); Investigation (equal). **Francesca Piccapane:** Data curation (equal); Methodology (equal). **Giuseppe Procino:** Writing‐review & editing (equal). **Martino Pepe:** Resources (equal). **Mara Piccolo:** Resources (equal). **Piero Guida:** Data curation (lead). **Nicoletta Resta:** Data curation; Investigation (equal). **Stefano Favale:** Writing‐review & editing (equal). **Maria Svelto:** Funding acquisition (lead); Writing‐review & editing (equal). **Monica Carmosino:** Conceptualization (equal); Formal analysis (supporting); Funding acquisition (supporting); Project administration (lead).

## Supporting information

Appendix S1

## Data Availability

The data that support the findings of this study are available from the corresponding author upon reasonable request.
