# Supplementary material for: Pro‐inflammatory cytokines as emerging molecular determinants in cardiolaminopathies
Source: J Cell Mol Med. 2021 Nov 12;25(23):10902–15. doi: 10.1111/jcmm.16975 (PMC8642682; doi:10.1111/jcmm.16975)
Supplement: Supplementary file 1 — Appendix S1 [file JCMM-25-10902-s001.docx]

**Supplementary Methods**

*Clinical and instrumental analysis.*

The inclusion/exclusion criteria were: 1) Proband or family member carrying a pathogenic or potentially pathogenic LMNA gene variant; 2) availability to provide written informed consent and to the processing of personal data. The exclusion criteria as follows: 1) Proband harbouring double mutations including both pathogenetic or potentially pathogenetic LMNA mutant and associated with pathogenetic or potentially pathogenetic variant in another gene related to cardiomyopathies; 2) patient not available to provide written informed consent and to the processing of personal data; 3) patients under the age of 18. Relatives not carrying the pathogenic or potentially pathogenic LMNA variant and with normal clinical and instrumental evaluations were considered control subjects. All subjects underwent clinical work-up, including medical history, physical examination, 12-lead electrocardiogram (ECG), transthoracic echocardiography, and 24-hour ECG monitoring and exercise testing. Where appropriate, patients without contraindications, such as pacemaker, defibrillator, or severe claustrophobia, underwent cardiac magnetic resonance imaging.

*Cytokine/chemokine assay.*

Complete panel screened for the expression of the following cytokines/chemokines: IL-1Ra, IL-1β, IL-2, IL-4, IL-5, IL-6, IL-7, IL-8, IL-9, IL-10, IL-12(p70), IL-13, IL-15, IL17A,eotaxin, FGF basic, G-CSF, GM-CSF, IFN-γ, IP-10, MCP-1 (MCAF), MIP-1a, PDGF-bb, MIP-1b, RANTES, TNF-a and VEGF. Fluorescence was quantified using a BioPlexMagpix Multiplex Reader (Bio-Rad Laboratories, Hercules, CA, USA). Each sample was analyzed in triplicate and the data automatically analyzed and processed using Bio-Plex Manager 6.0 software (Bio-Rad Laboratories, Hercules, CA, USA).

*Serum exosome preparation and analysis.*

Exosome pellets were lysed in RIPA buffer (150 mM NaCl, 10 mM Tris, pH 7.2, 0.1% SDS, 1% Triton X-100, 1% deoxycholate, 5 mM EDTA). Equal volumes of exosomes samples, diluted in Laemmli’s buffer with 10% beta-mercaptoethanol and heated for 10 min at 95°C, were resolved on 10% Mini-PROTEAN® TGX Stain-Free™ Precast Gels (Bio-Rad) and analyzed by Western blotting using the following antibodies: rabbit anti-Hsp70 (dil 1:1000, System Biosciences), rabbit anti-CD81 (dil. 1:0000, System Biosciences), rabbit anti-CD9 (dil. 1:0000, System Biosciences) and mouse anti-GAPDH (1:5000, Sigma). Densitometry was performed using the Image Lab™ software (Bio-rad) of ChemiDoc™ (Bio-Rad) imaging system, after normalization for the total protein content using the Stain-Free™ technology (Bio- Rad) according to manufacturer’s instructions.

*Cell culture and Western Blotting*

72 h after transfection HEK293 cells were lysed in RIPA buffer (150 mM NaCl, 10 mM Tris, pH 7.2, 0.1% SDS, 1% Triton X-100, 1% deoxycholate, 5 mM EDTA). 5 μg of each supernatant were separated by standard SDS-PAGE using Mini-PROTEAN® TGX Stain-Free™ Precast Gels Bio-Rad and analyzed by Western blotting using the following antibodies: rabbit anti-Hsp70 (dil. 1:1000, System Biosciences) and rabbit anti-m-cherry (dil 1:1000, Abcam). Densitometry was performed using the Image Lab™ software (Bio-rad) of ChemiDoc™ (Bio-Rad) imaging system, after normalization for the total protein content using the Stain-Free™ technology (Bio-Rad) according to manufacturer’s instructions.
